# Supplementary material for: Chromothripsis during telomere crisis is independent of NHEJ, and consistent with a replicative origin
Source: Genome Res. 2019 May;29(5):737–49. doi: 10.1101/gr.240705.118 (PMC6499312; doi:10.1101/gr.240705.118)
Supplement: Supplemental Material [file supp_gr.240705.118_Supplemental_file_1.zip › contigs/annotated_contigs/DB73/contig.2.DB73_length_571_mean_cov_4.72854640981.docx]

**DB73_length_571_mean_cov_4.72854640981**

```
TATTGTTAGGAATGTACCATAGTAAGATATATTAAATTAAGTATGGAAAAAAAGATGGCATCCTAGGGACTATCCCAAGGTAGGTTAAA
 >chr4:91647550-91647874 + E=2e-183
CTTTAGAGATACATGAATTTAAAATGGCAGTTCAGTCTTGTTTGGGATCAAATGTTTATTTTTTCCAATATGGCAGCATTTTTCAACTT

TGTTAGTATTGGAGCCCATTCTTTCAATTAATTAAAAACGATCTGCCCTGAAAGATAGTCTAAGCCCCACATACATACTCACTTCTCTA

TTCCCCCACACTATGGTTCAGTTCCTGCTAATCTCAAATTTACTGTTTAAAA|ACCAT|AATGACTTCCAGCTCCATCCCTGTTGTTAC
                                                     >chr4:91653964-91654216 + E=6e-140
AAATGATAGCATCTAATTCTTTTTTATGGCTGAATAGTGTTCATTGTGTATATGTATCACATTTTCTTAATCCATTCATCTGTTGATAG

TCACTTGGATTATTTGCAAATCTTGACTGCTACGAATAGTGCTACAGTAAACATGGGAGTGCAGATATCTCTTCAATACACTAATTTCC

CTTCTTTTGGGAATATATCTAGCAGTGGGATTGCTGGAT
```
